# Efficacy and Safety of Combined Brain Stereotactic Radiotherapy and Immune Checkpoint Inhibitors in Non-Small-Cell Lung Cancer with Brain Metastases

**DOI:** 10.3390/biomedicines10092249

**Published:** 2022-09-10

**Authors:** Judith Porte, Caroline Saint-Martin, Thomas Frederic-Moreau, Marie-Ange Massiani, Laurence Bozec, Kim Cao, Pierre Verrelle, Joelle Otz, Eric Jadaud, Mathieu Minsat, Adriana Langer, Nicolas Girard, Gilles Créhange, Arnaud Beddok

**Affiliations:** 1 Radiation Oncology Department, Institut Curie, PSL Research University, 75005 Paris, France; 2 Department of Biostatistics, Institut Curie, 92210 Saint Cloud, France; 3 Department of Thoracic Oncology, Institut Curie, 92210 Saint-Cloud, France; 4 Department of Medical Oncology, Institut Curie, 92210 Saint-Cloud, France; 5 Department of Imaging, Institut Curie, 92210 Saint-Cloud, France; 6 Department of Thoracic Oncology, Institut du Thorax-Site Curie, 75005 Paris, France; 7 Institut Curie, PSL Research University, University Paris Saclay, Inserm LITO U1288, 91401 Orsay, France

**Keywords:** stereotactic radiotherapy, immunotherapy, non-small cell lung cancer, brain metastases

## Abstract

Background: To analyze the outcomes of patients with brain metastases (BM) from non-small cell lung cancer (NSCLC) treated with immunotherapy (IT) and stereotactic radiotherapy (SRT) and to study the impact of the sequence between the two modalities. Methods: The authors reviewed the records of 51 patients with 84 BM from NSCLC treated at Institut Curie with IT and SRT. BM were categorized into three groups: ‘SRT before IT’, ‘concurrent SRT and IT’, and ‘SRT after IT.’ Regional progression-free interval (R-PFI) and overall survival (OS) were estimated using the Kaplan–Meier method. Results: After a median follow-up from SRT of 22.5 months (2.7–47.3), the 1-year and 2-year OS were 69.7% (95%CI [58.0–83.8]) and 44.0% [30.6–63.2], respectively. Concerning distant intracranial control, the 1-year and 2-year R-PFI were 40.1% [30.1–53.3] and 35.2% [25.1–49.4], respectively. Moreover, one-year R-PFI in ‘SRT before IT’, ‘concurrent SRT and IT’, and ‘SRT after IT’ groups were 24.1%, 49.6%, and 34.2%, respectively (*p* = 0.094). The type of therapeutic sequence did not appear to impact the risk of brain necrosis. Conclusions: The concurrent administration of SRT and IT appeared to offer the best locoregional control, without increasing the risk of toxicity, compared to patients treated with SRT before or after IT.

## 1. Introduction

Lung cancer remains the leading cause of brain metastases (BM) (responsible for 40–50% of all BM) [1]. The role of whole-brain radiotherapy (WBRT) has been challenged over time, especially for cancers in the late refractory setting. Moreover, WBRT consistently induces neurological toxicity, particularly neurocognitive decline [2]. Compared with WBRT, both stereotactic radiosurgery (SRS), in which the radiation is delivered in one session, and fractionated stereotactic radiotherapy (SRT) have proven their interest in oligometastatic patients, as they reduce neurological toxicity without loss of overall survival (OS) [3,4,5]. In fact, SRS and SRT have become the reference treatments, routinely used for patients with less than five BM and with a survival expectancy of more than 3–6 months [6,7,8]. In addition, over the past decade, immunotherapy (IT) with PD-1 pathway inhibitors has led to significant progress in the management of advanced non-small cell lung cancer (NSCLC), with major improvements in progression-free survival (PFS) and OS [9,10,11,12,13,14]. A phase-2 study suggested that pembrolizumab was active against BM from NSCLC, with PD-L1 expression ≥ 1% [15]. Thus, the question of the interaction between IT and SRT is increasingly being raised. In a recent meta-analysis, Yang et al. reported the results of 19 studies including patients with BM from NSCLC treated with RT with or without IT [16]. Most of the included studies have shown that the combination of RT and IT increased OS and regional PFS compared to RT alone, without inducing more brain necrosis (BN). Moreover, Lee et al. recently reported the outcomes of 77 patients with BM from NSCLC who received IT alone (*n* = 26), IT with concurrent Gamma Knife radiosurgery (GKS) within 14 days (*n* = 24), or IT with non-concurrent GKS (*n* = 27) [17]. OS, regional, and local PFS were higher in the group receiving IT and GKS compared to the “IT alone” group. Similarly, Guénolé et al. reported the outcomes of 99 patients (171 BM) who received SRT and concurrent systematic treatment (including 30 patients receiving IT) and 95 patients (131 BM) who received SRT alone without concurrent systemic treatment [18]. The patients who received concurrent IT had better 1-year local control, OS, and regional PFS compared to patients who did not (*p* < 0.05). Thus, these different studies showed that the combination of SRT and IT allowed higher local control and regional PFS than SRT alone [16]. The objective of the present study was to retrospectively assess the outcomes and toxicity of the combined use of IT and SRT in a relatively large cohort of patients with BM from NSCLC and to propose an optimal therapeutic sequence between IT and SRT.

## 2. Materials and Methods

### 2.1. Patient Selection

**Figure 1:**
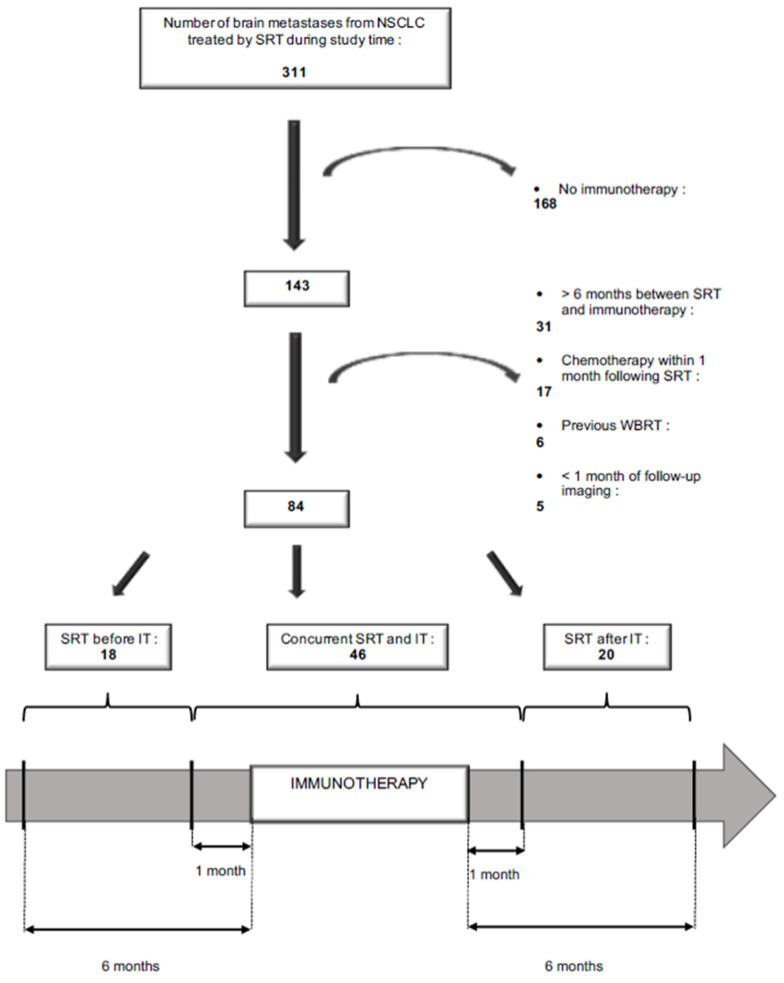
Flow-chart.

Figure 1 explains how patient selection was performed. Among the 266 patients with a total of 311 brain metastases (BM) from NSCLC treated in Institut Curie between February 2015 and December 2019 with stereotactic radiotherapy (SRT), 106 (143 BM) received immunotherapy (IT). Patients were retained for inclusion if they had received SRT in a period running from six months prior to IT to six months after IT. They were then categorized into three groups based on the relative timing of the therapies. In the ‘SRT before IT’ group, SRT was completed at least one month before the start of the IT treatment. In the ‘concurrent SRT and IT’ group, SRT was done within 1 month of IT. In the ‘SRT after IT’ group, SRT was done at least one month after the last course of IT. Patients were excluded if they had been treated with chemotherapy or targeted therapy during the month following SRT or if they had had previous WBRT. BM were present at the diagnosis of NSCLC, or occurred during cancer follow-up, and were confirmed by a brain MRI or pathologically. They could either be intact or previously resected (SRT of the surgical bed). Treated lesions without at least one month of follow-up imaging were excluded. Patients who had not indicated their non-opposition to the use of data concerning them were also excluded. Decisions concerning systemic treatment with IT were validated by multidisciplinary staff, and decisions on treatment with SRT were made during Stereotactic Radiation staff. The chart review and the present study were submitted and approved by the Curie institutional ethical committee (DATA200277).

### 2.2. Radiation Therapy

For SRT planning purposes, all patients underwent simulation, which consisted of the fabrication of a personalized thermoplastic mask using the commercial stereotactic mask fixation system of Frameless BrainLab Bivalve, and a custom thermoformed mattress for immobilization in the supine position, followed by CT imaging without contrast enhancement. All patients were treated with Novalis STx. For non-resected lesions, macroscopic disease (gross tumor volume [GTV]) was contoured on thin-slice (1 mm) gadolinium-enhanced T1-weighted axial MRI sequences fused with planning CT. The microscopic extension (clinical target volume [CTV]) was defined as equal to the GTV. For post-operative lesions, the CTV was obtained by adding 1 mm to the contouring of both the edge of the resection cavity and the residual enhanced tissue for subtotal resections. The CTV could also include 5 mm along the dura underlying the bone flap to account for microscopic disease extension in cases with preoperative dural contact, and a margin of less than 5 mm into the adjacent sinus when preoperative venous sinus contact was present. For all lesions, the provisional target volume (PTV) was generated by expanding the CTV by 1 mm in all directions. Patients with lesions of up to 20 mm were treated with single-fraction SRT, while larger lesions or small lesions located near or in eloquent areas (i.e., motor, speech, and brainstem) received multi-fraction SRT. In patients undergoing single-fraction SRT, the doses ranged from 15 to 18 Gy. For lesions treated with multi-fraction SRT, a dose from 18 to 27 Gy in 3 to 5 fractions was delivered. Doses were generally prescribed at the 80–85% isodose line and delivered using dynamic arcs. Cone-beam CT and ExacTrac image-guided systems were used to ensure accurate patient positioning. In the present study, several indices were used to assess the treatment plan quality. The Conformity Index [19], Gradient Index [20], and Homogeneity Index [19] should be between 0.9 and 2, between 3 and 6, and ≥1.25, respectively. The dose constraints for the normal brain were as follows: for one fraction, the volume receiving 12 Gy or more should be less than 8.4 cc (V12Gy < 8,4 cc), V10Gy < 10.4 cc, V8Gy < 12.4 cc, for 3 fractions and exclusive irradiation: V19.6-GTV < 10 cc, V23.1-GTV < 7 cc; for 3 fractions and post-operative irradiation: V24Gy < 16.8 cc, for 5 fractions: V28.8Gy < 7 cc, V20Gy < 20 cc.

### 2.3. Immunotherapy

The immune checkpoint inhibitors (ICI) used were intravenous nivolumab (240 mg every two weeks), pembrolizumab (200 mg every three weeks), durvalumab (10 mg/kg every two weeks), and atezolizumab (1200 mg every three weeks). Nivolumab, pembrolizumab, and atezolizumab could be used either as a first metastatic line or as a late line treatment. Durvalumab was used as a consolidation treatment after curative radio-chemotherapy.

### 2.4. Follow-Up

Each follow-up visit included a clinical examination. To evaluate the intracranial disease response and detect radionecrosis, a brain MRI was conducted six weeks after SRT, then every three months during the first two years, and then every six months thereafter or until death. Neurologic toxicity was recorded at each patient visit during the SRT treatment as well as in follow-up clinical evaluations and graded according to the Common Terminology Criteria for Adverse Events (CTCAE v5.0). All patients with data recorded in earlier versions of the CTCAE were reclassified according to version 5.0. Patients with significant or symptomatic edema were treated with corticosteroids. Radionecrosis was confirmed by pathologic examination of resected tissues, with lesions consistent with the effect of treatment with SRT, or attested by MRI imaging (using T2/FLAIR and Gadolinium-enhanced T1 sequences and sometimes with the need to refer to perfusion MRI), reviewed by a radiologist specialized in neuroradiology (AL). Brain progression and radionecrosis were assessed following the recommendations of the Response assessment in neuro-oncology (RANO) group [21]. The diagnosis was also in favor of radionecrosis if the edema resolved on steroids, with no subsequent further progression.

### 2.5. Statistical Analysis

Follow-up was extended from the date of SRT initiation to the date of the last news (i.e., last encounter with medical care). The median follow-up was estimated using the inverted Kaplan–Meier method. For baseline characteristics, qualitative data are presented as numbers and percentages, and continuous variables are presented as means and standard deviations or medians with minimum and maximum values (or interquartile range). The Chi 2 test or Fisher test was used to analyze the contingency tables.

Overall Survival (OS) was defined as the time between the date of the first SRT initiation and the date of death for deceased patients. Patients still alive were censored on the date of their last news. Local Progression-Free Interval (L-PFI) extended from the date of SRT initiation to the date of local progression of BM previously treated with SRT and visible on follow-up radiographic imaging, or death from any cause. Regional Progression-Free Interval (R-PFI) extended from the date of SRT initiation to the earliest date of regional progression (new BM or progression of a BM other than the one treated, and visible on follow-up intracranial radiographic imaging). In the absence of any event, the patients were censored on the date of their last news. Survival distributions were calculated using the Kaplan–Meier method and compared using the Log-rank test. It should be noted that among the 51 patients, 4 were irradiated more than once while they belonged to different groups. We therefore studied OS for the 51 patients, and R-PFI and L-PFI for the 84 metastases. L-PFI and R-PFI were calculated for the whole cohort after separating the three described groups for comparison: ‘SRT before IT,’ ‘concurrent SRT and IT,’ and ‘SRT after IT.’

Univariate and multivariate Cox regression models with the Cox stepwise procedure were used to assess the relative influence of prognostic factors. The following prognostic factors were assessed: sex, ECOG Performance Status (0/1 vs. 2/3), age at SRT (≤60 years vs. >60 years), line of immunotherapy (consolidation vs. 1st line vs. 2nd line or beyond), extra-cranial disease control at the time of SRT (yes vs. no), surgery (yes vs. no), number of fractions (1–2 vs. >2), and timing of treatment (concurrent SRT and IT versus SRT before or after IT).

Variables with a *p* value ≤ 0.20 in univariate analyses were included in the multivariate analyses. The added value of each variable in the Cox model was determined using a likelihood ratio test. A *p* value ≤ 0.05 was considered statistically significant. R 3.6.3. software was used for the analyses (http://cran.r-project.org (accessed on 17 February 2020)).

## 3. Results

### 3.1. Patients’ Characteristics

Fifty-one patients who received SRT between February 2015 and December 2019 for 84 BMs were included (Figure 1). The patients’ characteristics are shown in Table 1. The median age at BM diagnosis was 63.7 years (range, 46.5–91.7). Thirty-seven patients (72.5%) had adenocarcinoma. All patients with adenocarcinoma had at least immunochemistry analysis to assess for *Anaplastic lymphoma kinase* (*EML4/ALK)* rearrangement and real-time Polymerase Chain Reaction (PCR) assay with high resolution melting (HRM) to assess mutation on *Epidermal Growth Factor Receptor* (*EGFR)*, *V-Raf murine sarcoma viral oncogene homolog B (BRAF),* and *V-Ki-ras2 Kirsten rat sarcoma viral oncogene homolog* (*KRAS*). From 2016, for patients without mutations found (22/37), next-generation sequencing was performed. For programmed cell death ligand 1 (PDL1), a qualitative immunohistochemistry assay was performed using rabbit monoclonal anti-PD-L1 clone SP263. Eight patients (15.7%) were on consolidation IT at the time of SRT (“Durvalumab” group), 17 patients (33.3%) were on first-line IT, and 26 patients (51.1%) were treated with IT as a 2nd line or beyond. The median duration of IT was 4.9 months (range, 0–44.4). The BM and SRT characteristics are summarized in Table 2. Overall, 76 (90.5%) lesions were intact, and 8 (9.5%) had previously been resected. Most lesions were treated with either 15 to 21 Gy in a single fraction (56.0%) or 18 to 27 Gy in three fractions (41.8%). Among all treated lesions, 53.6% were treated in the context of isolated intracranial progression, while 46.4% were treated in the context of uncontrolled extracranial disease. Table S1 summarizes the PD-1 pathway inhibitor regarding the timing of immunotherapy and radiotherapy.

The ‘SRT before IT’ group included brain metastases (BM) from patients never pre-treated with IT: 13/18 BM of patients who have been progressed from first line chemotherapy and then received SRT before starting IT as second line therapy, and 5/18 BM discovered during follow-up after a curative treatment of lung cancer, or discovered at the diagnosis of the NSCLC and for whom SRT was therefore completed prior to initiating metastatic first-line IT. The ‘SRT after IT’ group included BM from patients who already received IT: 7/20 BM from patients who progressed after consolidation IT (“Durvalumab” group) and 13/20 BM from patients who received SBRT after IT as the 1st metastatic line or more. The ‘Concurrent SRT and IT’ group included BM of patients treated with IT as a metastatic line of treatment (43/46) or as a consolidation treatment: “Durvalumab” group (3/46).

Moreover, there were a few patients with “targetable” mutations in our cohort. The “*EGFR* mutated patients” remained on targeted therapies rather than immunotherapy. For the patient with a *Mesenchymal Epithelial Transition Factor Receptor* (*MET)* activating mutation, pembrolizumab was prescribed as a first-line therapy prior to receiving next-generation sequencing (NGS) results. IT was concomitant with SRT. He then received crizotinib as a second-line therapy. For the patient with a *REarranged during Transfection* *(RET)* translocation, niboluùab was prescribed as a second-line concomitant with SRT. The patient received pralsetinib as a third-line therapy. For the patient with a *human epidermal growth factor receptor 2 (HER2)* activating mutation, trastuzumab was prescribed as a second-line therapy. The patient received atezolizumab as the fourth line of treatment, concomitant with SRT after chemotherapy.

### 3.2. Locoregional Control and Overall Survival

**Figure 2:**
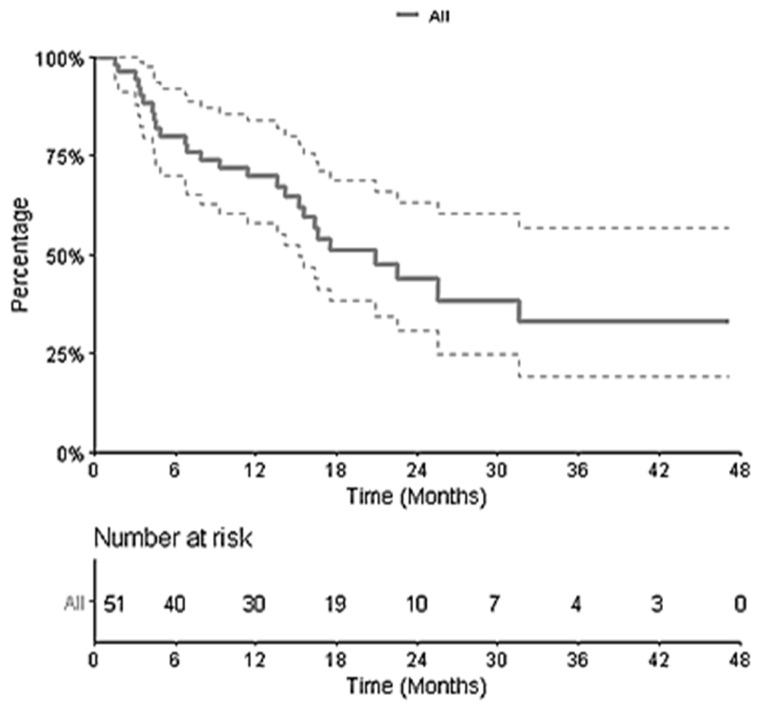
Kaplan–Meier Curve of Overall Survival for the 51 patients.

After a median follow-up from SRT of 22.5 months (2.7–47.3), for all 51 patients, the 1-year and 2-year OS were 69.7% (95% CI [58.0–83.8]) and 44.0% (95% CI [30.6–63.2]), respectively (Figure 2). The median OS was 18 months. In the univariate analysis, no variable other than ECOG Performance Status had a significant impact on OS.

**Figure 3:**
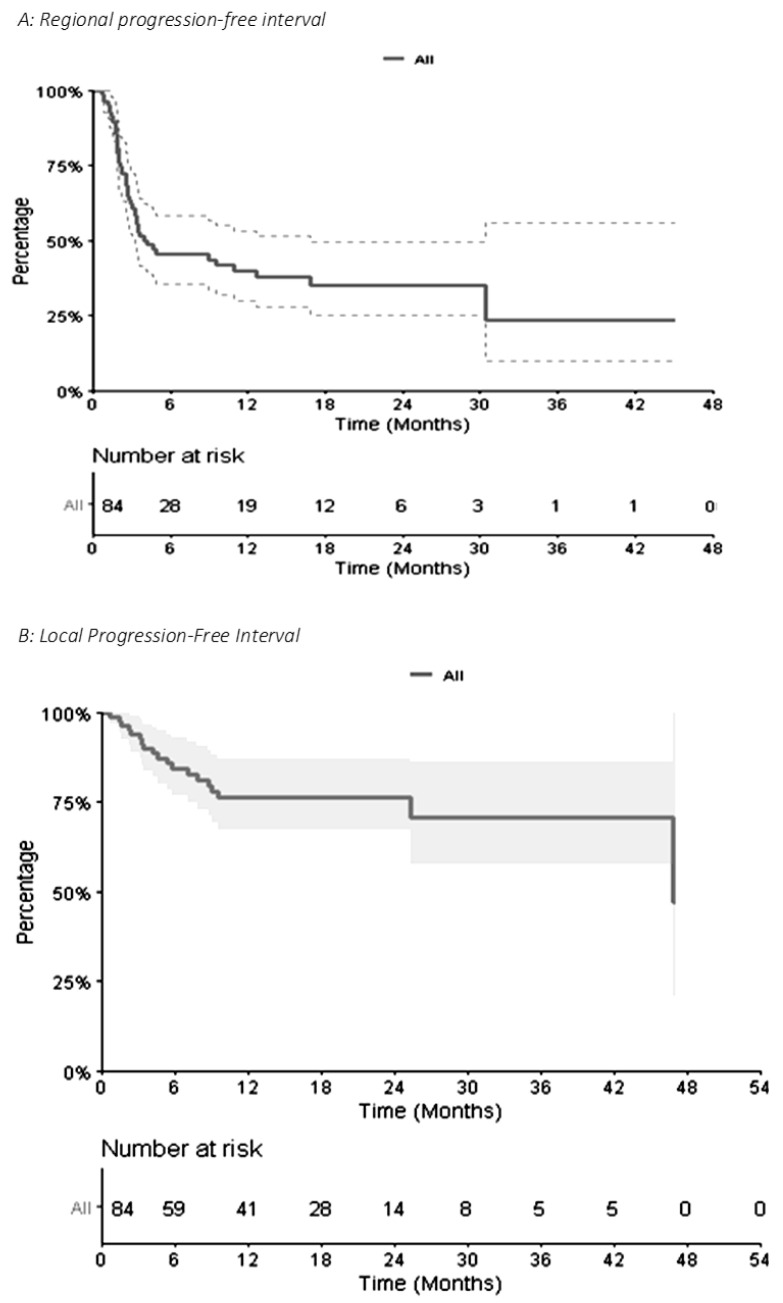
Kaplan–Meier Curve of Regional Progression-Free Interval (**A**) and Local Progression-Free Interval (**B**) for 84 metastases.

**Figure 4:**
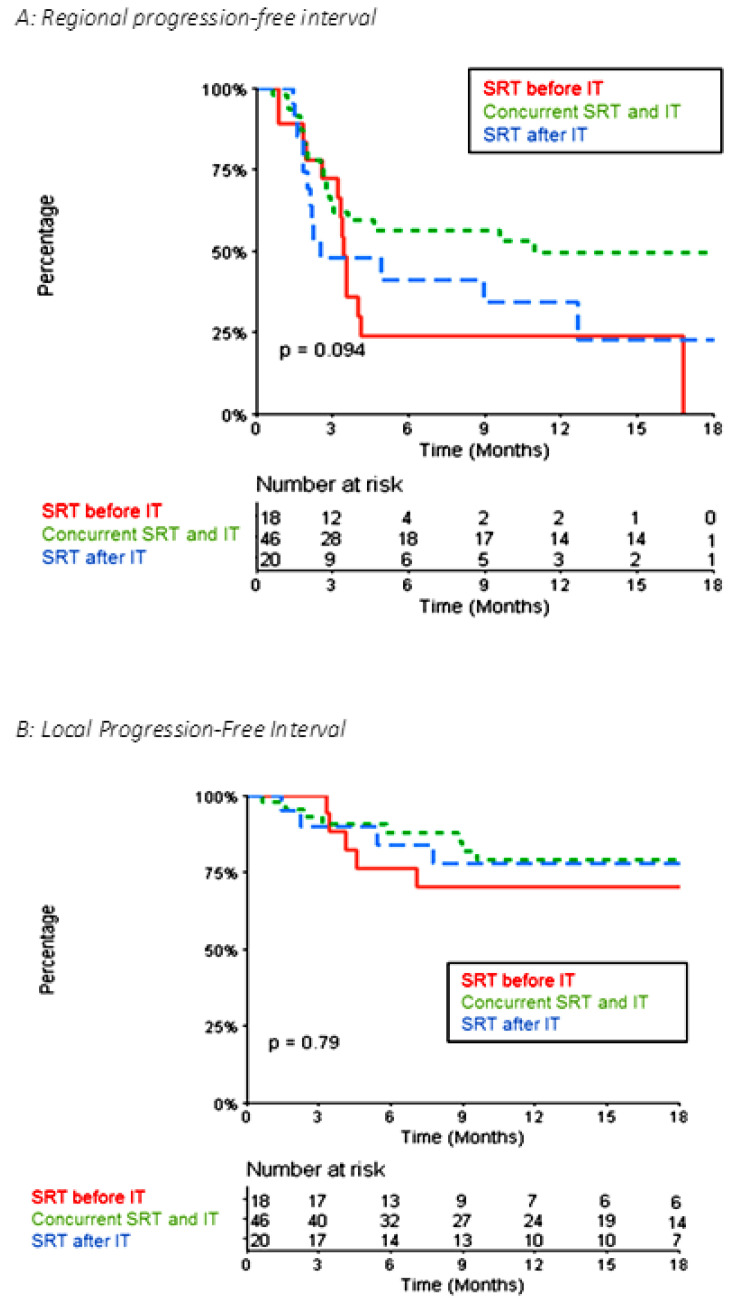
Kaplan–Meier Curve of Local Progression-Free Interval (**A**) and Local Progression-Free Interval (**B**) comparing the ‘concurrent SRT and IT,’ ‘SRT before IT,’ and ‘SRT after IT’ groups for the 84 metastases.

Concerning distant intracranial control, for the 84 metastases, the 1-year and 2-year R-PFI were 40.1% (95% CI [30.1–53.3]) and 35.2% (95% CI [25.1–49.4]), respectively (Figure 3A). The median time to local relapse was 4.14 months. The 1-year and 2-year L-PFI were both 76.4% (95% CI [67.0–87.0]) (Figure 3B). R-PFI tended to be longer in patients treated with ‘concurrent SRT and IT’ than in those treated with SRT before or after IT (Table S2). Indeed, the 1-year R-PFI in ‘concurrent SRT and IT,’ ‘SRT before IT,’ and ‘SRT after IT’ was 49.6% (95% CI [36.1–68.3]), 24.1% (95% CI [10.3–56.3]), and 34.2% (95% CI [17.6–66.3]), respectively (*p* = 0.094) (Figure 4A). The L-PFI was similar in the three groups. One-year L-PFI was 78.9% (95% CI [66.8–93.3]) in the ‘concurrent SRT and IT’ group vs. 70.1% (95% CI [51.2–96.0]) in the ‘SRT before IT’ group vs. 77.8% (95% CI [60.6–99.8]) in the ‘SRT after IT’ group (*p* = 0.79) (Figure 4B).

**Figure 5:**
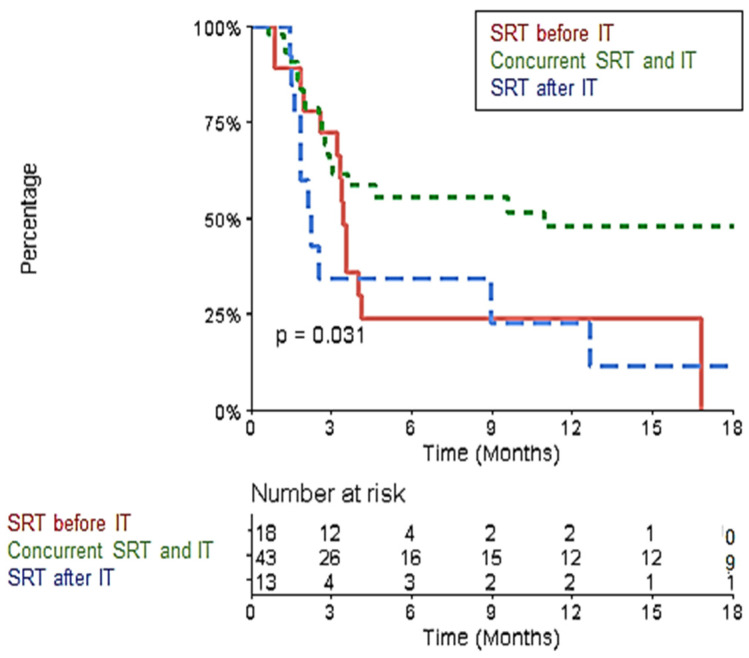
Kaplan–Meier Curve of Local Progression-Free Interval comparing the ‘concurrent SRT and IT,’ ‘SRT before IT,’ and ‘SRT after IT’ groups after excluding the “Durvalumab” group.

In the multivariate analysis, the R-PFI was significantly better in the “Durvalumab” group (Table S2). After excluding the 10 metastases belonging to this group, the 1-year R-PFI was significantly better in the ‘concurrent SRT and IT’ group than the 1-yeat R-PFI in the ‘SRT before IT’ and ‘SRT after IT’ groups: 48% (95% CI [34–67.8]) vs. 24.1% (95% CI [10.3–56.3]) vs. 22.8% (95% CI [7.4–70.1]), respectively (*p* = 0.031) (Figure 5).

### 3.3. Toxicity

#### 3.3.1. Acute Neurologic Toxicity

Radiation-induced acute neurologic toxicities are summarized in Table S3. Concurrent treatment did not appear to induce more acute neurologic toxicity (*p* = 0.66). No grade 4 or 5 toxicities were observed. One patient with five lesions treated in the ‘SRT after IT’ group experienced intracranial hypertension caused by SRT-induced edema, resulting in nausea, headache, vomiting, and a simple focal seizure of the left leg. High doses of IV corticosteroids and anti-epileptic drugs led to a rapid recovery. One patient in the ‘concurrent SRT and IT’ group experienced worsening of a pre-existing motor deficit of the left side of the body due to an SRT-related cerebral edema, which partially resolved after high doses of corticosteroids. Concerning a patient with three lesions treated in the ‘SRT before IT’ group, who had a grade 3 seizure, MRI showed that the symptoms were more likely due to regional progression than to the lesions treated with SRT. One patient in the ‘concurrent SRT and IT’ group experienced impaired speech after brain surgery, and the impairment persisted after SRT.

#### 3.3.2. Brain Radionecrosis

Among the 84 treated lesions, 11 cases of brain radionecrosis (BN) were observed at a median of 3.2 months (1.1–9.5) following the end of the SRT. The number of BN was not significantly different between the three studied subgroups. One patient in the ‘SRT after IT’ group experienced nausea, vomiting, headaches, and cerebellar syndrome, one patient in the ‘concurrent SRT and IT’ group had Wernicke aphasia, and the third patient belonging to the ‘SRT after IT’ group experienced headaches.

## 4. Discussion

Our retrospective series reports outcomes in 51 patients receiving SRT for 84 BM from NSCLC and treated with SRT and IT concurrently or non-concurrently. We found a trend toward better R-PFI in the ‘concurrent SRT and IT’ group than in the ‘SRT before or after IT’ groups. The type of therapeutic sequence did not appear to have an impact on acute neurologic toxicity or the incidence of BN.

It is now accepted that SRT may induce immunogenic cell death and stimulate the immune system [13,22,23,24]. Numerous pre-clinical studies have highlighted the mechanisms by which this interaction may occur: via the triggering of tumor antigen release, also via an increase in their cross-presentation, an increase in the expression of MHC Class 1 on the plasma membrane (making irradiated cells more recognizable by the immune system), the increased release of pro-inflammatory cytokines and DAMPs, stimulation of the recruitment of cytotoxic CD8+ T lymphocytes in the irradiated lesion’s microenvironment via dendritic cells, and increased cell expression of FAS [25]. When these above-described mechanisms are induced during combined SRT and IT, the immune-mediated anti-tumor activity may indeed be optimized. In pre-clinical studies, the choice of therapeutic sequence between SRT and IT remains controversial. In a murine model of weakly immunogenic breast cancer (TSA cell line), Dewan et al. showed that the best abscopal effect was obtained with a dose of 24 Gy in 3 fractions of 8 Gy (vs 1.20 Gy and 5.6 Gy) and when the anti-CTLA-4 antibody was administered over the days following irradiation, without exceeding a 4-day interval, beyond which the benefit of this combined treatment was no longer observed [26].

The essential part of the literature concerning the treatment of BM with combined IT and SRT mainly includes retrospective trials on melanoma patients and only a few on NSCLC patients. In a meta-analysis including a large majority of studies about patients with metastatic melanoma, Lu et al. [27] found that concurrent IT with SRS, in comparison with non-concurrent IT, conferred a significant 12 months-OS benefit (OR = 1.74; *p* = 0.011), and comparable 12 months-local PFS (OR = 2.09; *p* = 0.154) and distant PFS (OR = 0.88; *p* = 0.839). In a second meta-analysis, Lehrer et al. [28] showed that co-administration of SRT and IT was associated with a potential gain in OS, improved regional intracranial control, and excellent local control without increasing brain radionecrosis rates. Still, in a population of patients with BM from melanoma, Kiess et al. [29] reported that patients treated with SRT during or before Ipilimumab had better OS and less regional recurrence than those treated with SRS after Ipilimumab. Murphy et al. [30] also found that melanoma patients receiving concurrent treatment, compared with those who received the two treatments sequentially, had significantly better R-PFS. Moreover, Ahmed et al. reported that six months of distant intracranial control in patients treated with SRT during or prior to anti-PD-1/PD-L1 therapy was significantly higher than that in patients who underwent SRT after antiPD-1/PD-L1 therapy [31].

In a recent meta-analysis, Yang et al. reported the results of 19 studies including patients with BM from NSCLC treated with RT with or without IT [16]. The combination of SRT and IT allowed for higher local control and R-PFI than SRT alone. The median OS observed in our study for the 51 patients (18 months) was similar to other previously published series [32]. Moreover, in our study, the RPF-I seemed to be better in the ‘concurrent SRT and IT’ group than in the ‘SRT before or after IT’ groups. This is consistent with other previously published series (Table 3) [32,33,34,35,36,37]. Schapira et al. demonstrated that patients treated with concurrent SRT and IT had lower rates of distant brain failure than did patients treated with SRT before or after IT [32]. One-year-distant brain control was 61.5%, 34.2%, and 0% in the ‘concurrent,’ ‘SRT before,’ and ‘SRT after’ groups, respectively (*p* = 0.042). In another study evaluating the outcomes of 51 patients with BM from NSCLC, Shepard et al. reported for the ‘IT and SRT concurrent group,’ one-year R-PFS of 47.5%, which is similar to our study (49.6%) [36]. Chen et al. also found that concurrent therapy predicted a reduced probability of the subsequent development of more than three BM [34]. Despite the wide range of symptoms considered for neurological toxicity in our study (Table S3), neurological toxicity did not appear to increase in the concurrent group, which is consistent with data in the literature.

The number of patients included in our series was relatively small compared with the other mentioned studies. Actually, we voluntarily limited the time between SRT and IT to six months in order to really consider the two treatments combined and to explore a potential interaction (Figure 1). Moreover, concurrence was defined as the initiation of SRT within one month of IT. This was based on an approximation of the half-lives of the PD-1 pathway inhibitors used. This is consistent with other studies that stated that the patients received RT within 1 month before or after IT as the concurrent ‘RT + IT’ group [16], except Chen et al. [34] and Lee et al. [17], who defined the time interval as two weeks, and Imber et al. [38], who defined the time interval as two months. In addition, we excluded patients who had received chemotherapy within one month following SRT in order to be sure that regional outcomes would only be the result of the interaction between SRT and IT, and that chemotherapy had no impact on the intracranial response.

In addition, in our study, there was some heterogeneity among the patients, including their status and prognosis at the time of SRT, which correlated with the number of lines of systemic treatment previously received. In a multivariate analysis, the number of lines of immunotherapy was significantly associated with the risk of recurrence. Patients in the “durvalumab” group received brain SRT because they progressed only in this organ (oligometastatic). Therefore, they had a better prognosis than metastatic patients and could constitute a selection bias. We performed an analysis excluding patients in the “durvalumab” group. This is consistent with the sub-mentioned studies that did not include patients with “durvalumab.” After the exclusion of these patients, in a more homogeneous group of patients, regional control was significantly better in the concurrent group; this is similar to the few other studies on this topic (summarized in Table 3).

Finally, the concurrent combination of SRS and IT seems to increase the R-PFI of patients with BM from NSCLC. These results will certainly be confirmed and refined by the prospective studies currently ongoing on this topic (*NCT02858869, NCT02978404, NCT03955198, NCT03774732*).

## Figures and Tables

**Table 1:** Patient characteristics (*n* = 51).

	Number	%
**Sex**		
Female	20	39.2
Male	31	60.8
**Age at BM diagnosis (median, range)**	63.7 (46.5–91.7)	
**ECOG Performance status at SRT**		
0	18	35.3
1	29	56.9
≥2	4	7.8
**Smoking status**		
Active	26	51
Former	23	45.1
Never	2	3.9
**Type of PD-1 pathway inhibitor**		
Nivolumab	24	47.1
Pembrolizumab	17	33.3
Durvalumab	8	15.7
Atezolizumab	2	3.9
**Line of immunotherapy**		
Consolidation (“Durvalumab” group)	8	15.7
1	17	33.3
≥2	26	51.3
**Histology**		
Adenocarcinoma	37	72.5
Squamous cell carcinoma	11	21.5
Other	3	6
**^1^ Somatic Mutation**		
**KRAS**	19	37.3
**HER2**	1	2
**RET**	1	2
**MET**	1	2
None found	19	37.3
NA	10	19.6
Others ^2^	0	0
**^3^ PDL-1 status**		
≤1%	4	7.8
>1–49%	9	17.6
>50%	19	37.3
NA	19	37.3

Abbreviations: No, number; BM, brain metastasis; PD-1, Programmed cell death 1; ECOG, Eastern Cooperative Oncology Group; SRT, stereotactic radiotherapy; NA, non-available ^1^ Somatic mutations available for 41 patients. Among the 10 patients who did not have available molecular data, 7 had squamous cell carcinoma and 1 had neuroendocrine tumor. For these patients, molecular assessment was not provided. For the two other patients, no mutations were found on the trans-bronchial biopsies, and the sample on guided CT biopsy was insufficient for accurate molecular reanalysis; *KRAS*: V-Ki-ras2 Kirsten rat sarcoma viral oncogene homolog; *HER2*: human epidermal growth factor receptor 2; *MET*: mesenchymal epithelial transition; *RET:* REarranged during Transfection ^2^ Neuroblastoma-RAS (*NRAS*), V-Raf murine sarcoma viral oncogene homolog B (*BRAF*),Epidermal Growth Factor Receptor (*EGFR*), Anaplastic lymphoma kinase (*ALK*), ROS Proto-Oncogene 1, Receptor Tyrosine Kinase (*ROS1*), ^3^ programmed cell death ligand 1 (PDL-1) status available for 32 patients.

**Table 2:** Brain metastases and radiation therapy characteristics (*n* = 84).

	Number	%
**Timing of SRT**		
SRT before IT	18	21.4
Concurrent SRT and IT	46	54.8
SRT after IT	20	23.8
**IT–SRT interval**		
SRT before (months, [median, range])	3.5 (1–5.2)	
SRT after (months, [median, range])	2 (1.1–5.2)	
**Localization**		
Frontal	24	28.6
Temporal	6	7.1
Parietal	19	22.6
Occipital	14	16.7
Cerebellum	17	20.2
Brainstem	4	4.8
**Size, median** (mm, [median, range])	12 (3.5–50)	
**Extracranial disease control at the time of SRT**		
Yes	45	53.6
No	39	46.4
**Resected lesions**		
Yes	8	9.5
No	76	90.5
**Post-operative residue**		
Yes	6	75
No	2	25

Abbreviations: SRT: stereotactic radiotherapy; IT: immunotherapy.

**Table 3:** Outcomes of the combination of IT and intracranial SRT for patients with brain metastases from lung cancer.

Trial	Groups	Number Patient/BMs	Tumor Type	1-y LC	1-y Distant Brain Control	Median OS (Months)
Schapira et al. 2018 [33]	concurrent SRS/IT	37/85	NSCLC	100%	61.5%	17.6^+^ (all the 37 patients)
	SRS before IT			72.3% (*p* = 0.016)	34.2%	
	SRS after IT			100%	0% (*p* = 0.042)	
Chen et al. 2018 [35]	SRS without IT	260/623	NSCLC	82%	NA	12.9°
	concurrent SRS/IT		Melanoma	88%		24.7° (*p* = 0.002)
	non-concurrent/IT		RCC	79%		14.5°
Lanier et al. 2019 [36]	SRS without IT	271/NA	NSCLC	96%	66%	6.1+
	SRS with IT		Melanoma	91%	46% (*p* < 0.01)	15.9+ (*p* < 0.01)
Shepard et al. 2019 [37]	SRS without IT concurrent SRS/IT	51/137	NSCLC	85.2%	66.5%	15.9+
				100% (*p* = 0.31)	47.5% (*p* = 0.061)	
NA Singh et al. 2019 [38]	SRS and CT	85/531	NSCLC	NA	NA	11.6+
	SRS and IT					10 + (*p* = 0.23)
	concurrent SRS/IT					10+
	non-concurrent SRS/IT					12.1+
Present study	concurrent SRS/IT	51/84	NSCLC	78.9%	49.6%	18 (all the 51 patients)
	SRS before IT			70.1% (*p* = 0.79)	24.1% (*p* = 0.094)	
	SRS after IT			77.8%	34.2%	

Abbreviations: y: year, BM: brain metastases, m: months, OS: overall survival, SRS: stereotactic radiosurgery, SRT: stereotactic radiotherapy, IT: immunotherapy, conc, concurrent, NA: not available, °: OS defined from date of intracranial metastatic disease diagnosis to date of death or last follow-up, ^+^: OS defined from date of SRS to date of death or last follow up.

## Data Availability

Research data are stored in an institutional repository and will be shared upon request to the corresponding author.

## Supplementary Materials

The following are available online at https://www.mdpi.com/article/10.3390/biomedicines10092249/s1, Table S1: PD-1 pathway inhibitor regarding the timing of immunotherapy and radiotherapy. Table S2: Multivariate analysis for Regional Progression-Free Interval; Table S3. Symptoms of acute neurologic toxicity.
