# Supplementary material for: Efficacy and Safety of Combined Brain Stereotactic Radiotherapy and Immune Checkpoint Inhibitors in Non-Small-Cell Lung Cancer with Brain Metastases
Source: Biomedicines. 2022 Sep 10;10(9):2249. doi: 10.3390/biomedicines10092249 (PMC9496146; doi:10.3390/biomedicines10092249)
Supplement: Supplementary file 1 [file biomedicines-10-02249-s001.zip › biomedicines-1882049-supplementary.pdf]

Table S1: PD-1 pathway inhibitor regarding the timing of immunotherapy and radiotherapy

	Number	%
**SRT before IT**	18	21.4
Nivolumab	10	55.6
Pembrolizumab	7	38.9
Durvalumab	0	0
Atezolizumab	1	5.5
**Concurrent SRT and IT**	46	54.8
Nivolumab	27	58.7
Pembrolizumab	15	32.6
Durvalumab	3	6.5
Atezolizumab	1	2.2
**SRT after IT**	20	23.8
Nivolumab	6	30
Pembrolizumab	7	35
Durvalumab	7	35
Atezolizumab	0	0

Table S2: Multivariate analysis for Regional Progression-Free Interval

MULTIVARIATE				R-PFI	
**Factors**		**n**	**HR**	**CI**	**Pvalue**
**Sex**	Female	37	1		0.113
	Male	47	0.62	[0.34 ; 1.12]	
**Age at SRT**	< 60 y	26	1		0.163
	≥ 60 y	58	0.62	[0.32 ; 1.2]	
**Treatment**	Conc SRT - IT	46	1		0.114
	SRT before IT	18	1.57	[0.77 ; 3.2]	
	SRT after IT	20	2.33	[1.04 ; 5.24]	
**IT line**	1	28	1		0.006
	≥2	23	1.78	[0.86 ; 3.7]	
	Consolidation (“Durvalumab group”)	10	0.35	[0.1 ; 1.17]	

HR, Hazard Ratio; CI, confidence interval; SRT, stereotactic radiotherapy; IT, immunotherapy; y, years

Table S3. Symptoms of acute neurologic toxicity

Symptoms	Grade 1			Grade 2			Grade 3		
	SRT before IT	concurrent SRT and IT	SRT after IT	SRT before IT	concurrent SRT and IT	SRT after IT	SRT before IT	concurrent SRT and IT	SRT after IT
Fatigue	1	5	1	1		1			1
Headache		3	2		3	1			
Dizziness/ Imbalance	1	1			2				
Nausea/ Vomitting									1
Motor impairment								1	
Ataxia		1							
Seizure							2		1
Speech trouble		1							
Confusion syndrome								1	
